# Stress-Induced Accumulation of *DcAOX1* and *DcAOX2a* Transcripts Coincides with Critical Time Point for Structural Biomass Prediction in Carrot Primary Cultures (*Daucus carota* L.)

**DOI:** 10.3389/fgene.2016.00001

**Published:** 2016-01-29

**Authors:** M. Doroteia Campos, Amaia Nogales, Hélia G. Cardoso, Sarma R. Kumar, Tânia Nobre, Ramalingam Sathishkumar, Birgit Arnholdt-Schmitt

**Affiliations:** ^1^EU Marie Curie Chair, ICAAM – Instituto de Ciências Agrárias e Ambientais Mediterrânicas, IIFA-Instituto de Formação e Investigação Avançada, Universidade de ÉvoraÉvora, Portugal; ^2^Molecular Plant Biology and Biotechnology Division, Council of Scientific and Industrial Research–Central Institute of Medicinal and Aromatic Plants Research CentreBangalore, India; ^3^Plant Genetic Engineering Laboratory, Department of Biotechnology, Bharathiar UniversityCoimbatore, India

**Keywords:** *Daucus carota*, alternative oxidase, cell reprogramming, growth induction, chilling, *DcAOX1* gene characterization

## Abstract

Stress-adaptive cell plasticity in target tissues and cells for plant biomass growth is important for yield stability. *In vitro* systems with reproducible cell plasticity can help to identify relevant metabolic and molecular events during early cell reprogramming. In carrot, regulation of the central root meristem is a critical target for yield-determining secondary growth. Calorespirometry, a tool previously identified as promising for predictive growth phenotyping has been applied to measure the respiration rate in carrot meristem. In a carrot primary culture system (PCS), this tool allowed identifying an early peak related with structural biomass formation during lag phase of growth, around the 4th day of culture. In the present study, we report a dynamic and correlated expression of carrot *AOX* genes (*DcAOX1* and *DcAOX2a*) during PCS lag phase and during exponential growth. Both genes showed an increase in transcript levels until 36 h after explant inoculation, and a subsequent down-regulation, before the initiation of exponential growth. In PCS growing at two different temperatures (21°C and 28°C), *DcAOX1* was also found to be more expressed in the highest temperature. *DcAOX* genes’ were further explored in a plant pot experiment in response to chilling, which confirmed the early *AOX* transcript increase prior to the induction of a specific anti-freezing gene. Our findings point to *DcAOX1* and *DcAOX2a* as being reasonable candidates for functional marker development related to early cell reprogramming. While the genomic sequence of *DcAOX2a* was previously described, we characterize here the complete genomic sequence of *DcAOX1*.

## Introduction

Plant breeding makes use of i*n vitro* systems for plant propagation, but these systems are also ideal to isolate scientific questions related to stress responsiveness for later up-scaling of the knowledge to plant level. Especially, early molecular plant responses during cell reprogramming upon abiotic stress can easily be targeted (Arnholdt-Schmitt, 1993a; Arnholdt-Schmitt et al., 1995 and references in: Arnholdt-Schmitt, 2004; Arnholdt-Schmitt et al., 2006; Frederico et al., 2009b; Zavattieri et al., 2010). In recent years, considerable progress has been made regarding the development and isolation of stress tolerant genotypes by using *in vitro* techniques (Pérez-Clemente and Gómez-Cadenas, 2012). Phenotypic variability shown in *in vitro* culture systems is due to high genotype dependence, going from species level to the level of cultivar/variety and individual genotypes. It can vary between organs/tissues and developmental stages (Cardoso et al., 2010 and references therein). This variability in response, known as *in vitro* recalcitrance, could be described as varying capacity for plant cells to adapt to new environmental conditions, i.e., the capacity to develop and express new cell programs. This general capacity is important at plant level when environmental conditions are changing. For example, efficient transformation of trichoblasts (see Arnholdt-Schmitt, 2004) to fine root hairs is important under changing phosphorus availability in the soil to guarantee access to the nutrient. Plant adaptive plasticity was recently proposed as a new trait in plant breeding (Nicotra et al., 2010; Cardoso and Arnholdt-Schmitt, 2013), since it influences the stability of plant biomass and yield production. Plant research for robust phenotypes that show stability in growth performance is crucial, but also the most critical and most expensive step in breeding. Efficient marker systems and reliable screening tools that can assist in identifying and selecting superior robust genotypes with differential adaptive plasticity are still important bottlenecks (Arnholdt-Schmitt et al., 2014, 2015a).

In molecular plant breeding, candidate gene approaches for marker-assisted selection are considered a promising strategy (Collins et al., 2008). Good candidate genes for multi-stress tolerance and yield stability are genes involved in cell coordination and decision making in target cells. AOX is increasingly getting into the focus of research on stress acclimation and adaptation and seems to play a key role in regulating the process of cell reprogramming by ameliorating metabolic transitions related with the cellular redox state and flexible carbon balance (Arnholdt-Schmitt et al., 2006, 2015a; Rasmusson et al., 2009). AOX is supposed to provide the respiratory system with built-in flexibility regarding the degree of coupling between carbon metabolism pathways, electron transport chain activity, and ATP turnover (Vanlerberghe, 2013). For this reason, *AOX* genes were proposed and adopted as candidate genes for functional marker development related to multi-stress tolerance and plant adaptive plasticity (Arnholdt-Schmitt et al., 2006; Polidoros et al., 2009; Cardoso and Arnholdt-Schmitt, 2013). *AOX* genes were found to be differentially transcribed in various systems early during *in vitro* culture –induced morphogenic responses. This includes *de novo* growth from quiescent root phloem tissue (Campos et al., 2009) and somatic embryogenesis (Frederico et al., 2009a) in carrot and adventitious rooting in olive (Macedo et al., 2009; Macedo et al., 2012).

Nogales et al. (2013) developed calorespirometry as a new tool for breeding in a carrot *in vitro* PCS. This *in vitro* system, originated from quiescent secondary tap root phloem, was first established by Steward et al. (1952) and later proposed by Arnholdt-Schmitt (1999) as a mean for carrot yield prediction. Calorespirometry has been shown to be useful to accurately monitor temperature dependent growth performance in terms of metabolic rates, respiratory rates, efficiency of biomass acquisition, and growth rates over 21 days of *in vitro* cultures (Nogales et al., 2013). Those data showed a drastic increase in structural biomass formation until around the 4th day after inoculation during the lag phase of growth.

In this paper, we expanded the number of cultures tested by Nogales et al. (2013) and first demonstrate that the increase in structural biomass formation, showing an early peak during the lag phase of growth, is present in all the five primary cultures tested. We report that both carrot *AOX* genes, *DcAOX1*, and *DcAOX2a*, previously demonstrated as the ones with major expression in the PCS (Campos et al., 2009), showed increased levels of transcripts until the 4th day of culture and subsequent down-regulation before exponential growth starts. As a first attempt to transpose these findings to plant level, we also show an early transcript accumulation for both *AOX* genes in a chilling plant pot experiment prior to the induction of a specific *AFP*. This study identifies *DcAOX1* and *DcAOX2a* as being reasonable candidates for functional marker development on efficient cell reprogramming under changing environments in general. The isolation and characterization of the complete genomic sequence of *DcAOX1* is further reported.

## Materials and Methods

### Establishment of a Primary Culture System (PCS)

Ten weeks-old plants of *D. carota* L. cv. Rotin grown in pots, containing commercial soil mixture maintained under greenhouse conditions were used. Five explants from the secondary tap root phloem of each plant were inoculated per Erlenmeyer containing 20 mL of NL liquid medium (Neumann, 1966) supplemented with kinetin (1 mgL^-1^) and indoleacetic acid (2 mgL^-1^). The cultures were incubated under continuous rotation (90 rpm) and continuous light (95–100 μmol m^-1^ s^-1^, Philips) at 21°C and/or 28°C. During culture, tissue dedifferentiation and subsequent callus formation and growth is induced. After the lag-phase of 6–8 days exponential callus growth starts mainly as a result of cell division activity, and typically, callus continues to proliferate during 28 days in culture. At day 14, the linear phase of callus growth is running and a mixotrophic nutritional system is established (Arnholdt-Schmitt, 1999). Due to a supplementation of cytokinin in the culture medium, no organogenesis is initiated during the experiment. In cells of initial explants taken from secondary phloem of mature tap roots usually only carotene-containing chromoplasts appear to be present and neither chloroplasts nor other plastid structures were found. However, during the first 8 days in culture, restructuring of chromoplasts to chloroplasts is initiated via an intermediate state as amylo-chloroplasts (Kumar et al., 1983).

### Calorespirometry Measurements

In order to calculate specific growth rates (i.e., structural biomass formation rate, R_struct_biomass_) and efficiencies of biomass acquisition as described in Nogales et al. (2013), the respiratory metabolic heat rates and CO_2_ emission rates were measured in PCS by calorespirometry, at different time points. To confirm reproducibility of the early peak for structural biomass formation reported by these authors in two PCS, we additionally performed new measurements in three PCS growing at two different incubation temperatures (21°C and 28°C). The total of five PCS measurements are presented.

### *DcAOX1* and *DcAOX2a* Expression Analysis

#### *AOX* Response During Tissue Dedifferentiation and Callus Growth

We studied *DcAOX1* and *DcAOX2a* mRNA levels in an *in vitro* PCS by:

(i) Semi-quantitative PCR (RT-sqPCR) on both *AOX* genes, in order to shed light on transcript changes during the earliest events related to cell reprogramming and also in the later growth phase. Explants from 4 individual carrot plants (four biological replicates) grown at 21°C were collected at different time points: 0 h, 4 h, 8 h, 12 h, 36 h, 4, 8, 14, 21, and 28 days post inoculation (hpi/dpi). From 0 hpi until 4 dpi, 30 explants were taken per time point. For the remaining time points, a maximum of 15 explants were taken. Samples were collected as bulked samples. FW of each callus was also determined at 0, 4, 8, 12, 14, 18, 21, and 28 dpi.

(ii) Quantitative real-time PCR (RT-qPCR), to compare the transcript changes of *AOX* on PCS under two incubation temperatures (21°C and 28°C, as in see Calorespirometry Measurements). Explants from five individual plants (five biological replicates) were collected at 0 and 14 dpi (T0 and T14). Samples consisted of bulked samples of about 50 explants. The five plants used on expression analysis (*n* = 5) resulted from a previous selection of 12 individual plants based on their callus growth behavior under the two temperatures tested.

#### AOX Response to Chilling Exposure of Carrot Plants

Seeds of *D. carota* cv. Rotin were sowed in pots containing commercial soil mixture and maintained at controlled conditions for 1 month (23°C, 70–75% of air humidity and 16 h photoperiod with 200 ± 20 μmol m^-1^ s^-1^ light intensity).

Two chilling exposure (CE) experiments were conducted by:

(i) Semi-quantitative PCR (RT-sqPCR), to study the effect of CE on *AOX* expression of seedlings growing under controlled conditions (as described above), and exposed to 4°C for 5 days. Samples were collected at different time points: 0, 1, 2, 3, 4, and 5 dpce. Samples consisted of young leaves taken as bulked samples from three individual plants. Three bulked samples (biological replicates) were considered in a total of 54 plants.

(ii) Quantitative real-time PCR (RT-qPCR), to evaluate early *AOX* transcript levels on seedlings exposed to 4°C for 24 h. Samples were collected at 0 h, 10 min, 45 min, 4 h, 6 h, and 24 h post CE (mpce or hpce) and at 24 and 48 h after transferring the plants back to the initial growth conditions, as described above (recovery period). Samples consisted of young leaves taken from single plants. Four plants (four biological replicates) were considered per time point. Additionally, the expression of carrot antifreezing protein *(DcAFP)* was evaluated in this experiment at the referred time points.

#### Sample Processing

Total RNA from all samples was extracted using RNeasy Plant Mini Kit (Qiagen, Hilden, Germany), with on-column digestion of DNA using RNase-Free DNase Set (Qiagen, Hilden, Germany), according to manufacturer’s instruction. DNase-treated total RNA (1 μg) were reverse transcribed with random decamer primer (PCS experiments) or the oligo d(T) primer (CE experiments), using the RETROscript^®^ kit (Ambion, Austin, TX, USA) according to manufacturer’s instruction. The maximum number of time points chosen to collect plant material for RNA extraction and for growth curve analysis was restricted by nature of root sizes.

#### Expression Analyses

(i) RT-sqPCR: all RT-sqPCR experiments were performed using Ready-To-Go PCR Beads (GE Healthcare, Little Chalfont, England), 2 μL of cDNA (diluted 1:10) as template and 0.2 μM of each specific primer (**Table 1**). *DcEF1α* was previous selected (results not shown) as the reference gene for all RT-sqPCR experiments. PCR for *DcEF1α* and *DcAOX1* (for primers sequences see **Table 1**) was carried out for 32 cycles, each one consisting of 30 s at 94°C, 15 s at 60°C, and 15 s at 72°C. For *DcAOX2a* the PCR was of 35 cycles consisting in 30 s at 94°C, 30 s at 55°C, and 30 s at 72°C. An initial step at 94°C for 5 min and a final extension at 72°C for 5 min were performed in both cases. A previous experiment confirmed that disinfection did not have influence on *AOX* transcript levels in PCS (data not shown). RT-sqPCR products were analyzed by electrophoresis in 2% (w/v) agarose gel. For PCS, image analysis was carried out to normalize the expression level of *AOX* cDNA with the reference *DcEF1α* gene, by density band analyses using ImageJ 1.47v software^1^ (Schneider et al., 2012). The results were expressed as mean ± SE of four individual plants. The initial time (0 h) was used as calibrator and was set to 1.00 of RE. Differences between time points were examined by one-way ANOVA using the STATISTICA 8.0 statistical package (StatSoft Inc.).

**Table 1:** Primers used in RT-sqPCR and RT-qPCR.

Gene	[mRNA NCBI accession ID]	Primer sequence (5′→3′)		AS (bp)	*E* (%) (*r^2^*)	
					PCS	CE
*DcEF1α*	[GenBank:GQ380566]	Fw	TGGTGATGCTGGTTTCGTTAAG	75	97.0 (0.996)	97.7 (0.996)
		Rv	AGTGGAGGGTAGGACATGAAGGT			
*DcAOX1*	[GenBank:EU286573]	Fw	CTTCAACGCCTACTTCCTTG	196	99.2 (0.996)	87.7 (0.994)
		Rv	ATCTCGCAATGTAGAGTCAGC			
*DcAOX2a*	[GenBank:EU286575]	Fw	TCTTCAATGCTTTCTTTGTTCTT	200	92.9 (0.993)	87.7 (0.992)
		Rv	GACATCTTTTAGTTTGGCATCTTT			
*DcAFP*	[Genbank:AJ131340]	Fw	CGACAAGCAAGC TTTACT CCAA	80	-	94.1 (0.992)
		Rv	CGTCTGACACCCATGAGTCTGT			

*Amplicon size *(AS)*, primers efficiency *(E)*, and regression efficiency *(r2)* for the primary culture experiment *(PCS)*, and chilling experiments *(CE)*.*

(ii) RT-qPCR: Transcript abundances of *DcAOX1, DcAOX2a*, and *DcAFP* (**Table 1**) were determined by RT-qPCR on a 7500 Real Time PCR System (Applied Biosystems, Foster City, USA) using Maxima SYBR Green q-PCR Master Mix (Fermentas, ON, Canada). Reaction (15 μL) consisted in 2 μL of first-strand cDNA (previously diluted 1:10) and 0.3 μM of each specific primer. *DcEF1α* was selected for data normalization based on previous experiments involving 12 candidate genes (Campos et al., 2015; data for PCS not shown). Amplicons of all genes were previously confirmed by direct sequencing. Standard curves of a fourfold dilution series (1:1–1:125) (run in triplicate) of pooled cDNA from all samples were used for primer efficiency determination. Minus reverse transcriptase and no template controls were included to assess contaminations. RT-qPCR was performed for 40 cycles, each consisting of 15 s at 95°C followed by 1 min at 60°C. To analyze the dissociation curve profiles, an additional step at 95°C during 15 s was added, followed by a constant increase of temperature between 60 and 95°C. The 2^-ΔΔCT^ methodology (Livak and Schmittgen, 2001) was used to normalize expression data. Samples collected at initial times (0 h in CE or T0 in PCS) were used as calibrators.

For PCS experiment (i) and CE experiment (ii), a One-way analysis of variance (ANOVA) with Tukey’s post hoc test was used to search for significant differences in gene expression between time points. Regarding the PCS experiment at different temperatures (ii), differences in transcript levels between temperatures at T14 were analyzed by Student’s *t*-test (*n* = 5). A Pearson’s product–moment correlation (Zar, 2010) was used to compare normalized expression data of *DcAOX1 versus DcAOX2a*. All statistical analyses were performed using the STATISTICA 8.0 statistical package (StatSoft Inc., Tulsa, Ok, USA). Significance levels were set at *P* < 0.05.

### *DcAOX1* Gene Isolation

#### Plant Material

Seeds of *D. carota* L. cv. Rotin were germinated *in vitro* in MS basal media (Murashige and Skoog, 1962) and maintained under controlled conditions (25 ± 1°C, 16 h photoperiod with 34 μmolm^-2^s^-1^ light intensity). Eight-week-old *in vitro* grown seedlings were used for gDNA and total RNA extraction. For gene identification, mixtures of several plants were used; for complete gene isolation at gDNA and cDNA level, single plants were taken.

#### Identification of *DcAOX1*

gDNA extraction was performed using DNeasy Plant Mini Kit (Qiagen, Hilden, Germany) following the manufacturer’s instructions. For *DcAOX1* gene identification (previously named *DcAOX1a*, Costa et al., 2009), degenerate primers (*P1/P2*, annealing at 60°C for 2 min and extension at 72°C for 2 min, **Table 2**), designed based on *A. thaliana* (Saisho et al., 1997) were used. PCR was performed with Ready-To-Go PCR Beads (GE Healthcare, Little Chalfont, England) using 10 ng of gDNA as template and 0.2 μM of each primer.

**Table 2:** Primers used for *DcAOX1* gene isolation.

Primer name	Sequence (5′→3′)
*P1*	CTGTAGCAGCAGTVCCTGGVATGGT
*P2*	GGTTTACATCRCGRTGRTGWGCCTC
*VIAL 8*	GACCACGCGTATCGATGTCGACTTTTTTTTTTTTTTTTV
*VIAL 9*	GACCACGCGTATCGATGTCGAC
*DcAOX1Fw*	GCAAGTCACTCAGGCGCTTTG
*P6*	CGCGGAAGAAGGCACATGGCTGAATA
*DcAOX1R*	ATCTCGCAATGTAGAGTCAGCC
*DcAOX1_25Fw*	ATTTCTGGTACATTTTAGTTTTGA
*DcAOX1_1304Rev*	CATGGTTTGACGAGGGATTT
*DcAOX1_24Fw*	AAAATAACAATGATGATGACACG
*DcAOX1_1032Rv*	AACCAGAGATTCCTCCACTTCA


*V* = A, C, or G; *R* = A or G; *W* = A or T.

#### Isolation of *DcAOX1* Complete Sequence

To determine the 5′ and 3′ ends of the isolated *DcAOX1* fragment, 5′ and 3′ RACE-PCRs were performed. Total RNA was isolated using RNeasy Plant Mini Kit (20) (Qiagen, Hilden, Germany), with on-column digestion of DNA with the RNase-Free DNase Set (Qiagen, Hilden, Germany) according to the manufacture’s protocol.

For 3′RACE-PCR, single cDNA strand was produced using the enzyme RevertAid^TM^ HMinus M-MuLV Reverse (Fermentas, ON, Canada), with oligo (dT) primer VIAL 8 (Roche, Mannheim, Germany) (**Table 2**), according to the manufacturer’s instruction. For the synthesis of the second cDNA strand and subsequent 3′end amplification, the reverse primer *VIAL 9* (Roche, Mannheim, Germany) (**Table 2**) was used in combination with gene-specific forward primer (*DcAOX1Fw*, annealing at 55°C for 30 s, extension 72°C for 60 s, see **Table 2**) designed based on previously isolated *AOX1 P1/P2* sequence. One moicro liter of a 1:10 cDNA dilution of first strand PCR product was used as template for amplification.

To isolate the 5′ end, a cDNA library of *D. carota* L. cv. ‘Marktgaertner’ M853 (kindly provided by Dr. Bettina Linke, Humboldt University of Berlin, Germany) cloned into a Lambda gt22a phage vector (Invitrogen, Karlsruhe, Germany) was generated (Linke et al., 2003). 5′ RACE-PCR was carried out using 1 μL of cloned library as template and vector specific forward primer *P6* (**Table 2**), combined with a gene-specific reverse primer designed based on the sequence previously isolated with *P1/P2 (DcAOX1R*, annealing at 55°C for 30 s and extension at 72°C for 2 min, see **Table 2**).

RACE-PCRs were performed using 0.5 U of *Taq* DNA polymerase (Thermo Scientific, Wilmington, DE, USA) with 1X manufacturer supplied (NH4)_2_SO_4_ buffer, 1.5 mM MgCl_2_, 0.2 mM of each dNTPs (Fermentas, ON, Canada) and 0.2 μM of each primer.

For complete gene (cDNA) isolation, a gene-specific primer set (*DcAOX1_25Fw* and *DcAOX1_1304Rv*, annealing at 52°C for 20 s and extension at 72°C for 2 min, **Table 2**) was designed based on 5′ and 3′-UTR sequences previously isolated with RACE-PCRs. For gDNA complete gene isolation, another gene-specific primer set (*DcAOX1_24Fw* and *DcAOX1_1032 Rv*, annealing at 64°C for 30 s and extension at 72°C for 2 min, **Table 2**) was designed. Ten ng of gDNA and a 1:10 cDNA dilution from a single plant were used as templates. PCRs were performed using Phusion^TM^ High-Fidelity DNA Polymerase (Finnzymes, Espoo, Finland) according to the manufacturer’s instruction, using 0.2 μM of each specific primer. All PCR products were analyzed in 1.4% (w/v) agarose gel.

#### Cloning and Sequence Analysis

All PCR fragments were purified using GFX PCR DNA and Gel Band Purification Kit (GE Healthcare, Little Chalfont, England) according to the manufacture’s protocol, and cloned into pGEM^®^-T Easy vector (Promega, Madison, WI, USA). Reaction products were transformed to *E. coli* JM109 (Promega Madison, WI, USA) competent cells. Plasmid DNA was extracted from putative recombinant clones (Bimboim and Doly, 1979) and confirmed by using *Eco*RI restriction enzyme (Fermentas, ON, Canada). Selected recombinant clones were sequenced^2^ (Macrogen, Seoul, Korea) using T7 and SP6 primers (Promega, Madison, WI, USA). Sequence homology was confirmed in NCBI GenBank database^3^ (National Center for Biotechnology Information, Bethesda, MD, USA) using BLASTn and BLASTp algorithm (Karlin and Altschul, 1993).

SeqMan and EditSeq softwares (LASERGENE 7, GATC Biotech, Konstanz) were used to edit the obtained *AOX* sequence. Phylogenetic studies included data retrieved from public web-based databases, freely available (NCBI)^3^, Plaza^4^; e!EnsemblPlants^5^ and IPK Barley Blast Server^6^. Non-annotated *AOX* sequences used for phylogenetic studies were previously identified (Cardoso et al., 2015).

Sequences were aligned using MAFFT software^7^ (online version) under the model G-INS-1 (Slow; progressive method with an accurate guide tree), all other variables left as default. The best-fit model of amino acid replacement was selected by Akaike Information Criterion (AIC) according to the software ProtTest (Darriba et al., 2011). The selected model of protein evolution (probability of change from a given amino acid to another over a period of time, given some rate of change) was JTT+I+G [the JTT empirical model (Jones et al., 1992), considering an invariable fraction of amino acids (+I) and assigning each site a probability to belong to a different category of rate change (Darriba et al., 2011)]. Phylogenetic reconstruction was done through maximum likelihood (ML) as implemented in the software MEGA v 0.6, under the above referred model, and bootstrapped with 1000 replicates. The phylogenetic tree was rooted with *Chlamydomonas reinhardtii AOX1* sequence.

MITOPROT software (Claros and Vincens, 1996) was used to predict mitochondrial targeting sequence and cleavage site. Gene draw was performed in FancyGene 1.4 (Rambaldi and Ciccarelli, 2009). For intron identification, the Spidey software^8^ was used.

## Results

### Calorespirometry in Primary Cultures

**FIGURE 1:**
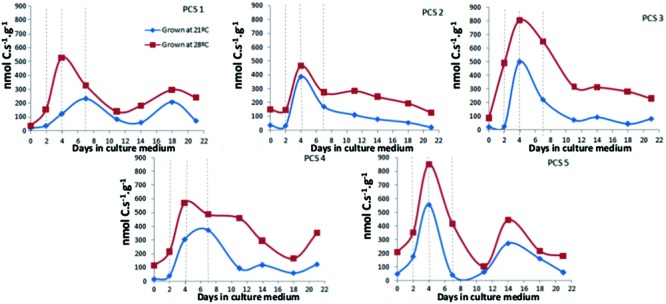
**R_structural biomass_ in callus grown at 21°C and 28°C in primary cultures during 21 days.** Each data point represents the average of three measurements performed using 200–300 mg of callus.

**Figure 1** shows the results for R_struct_biomass_ calculated from calorimetrically measured R_q_ and R_CO2_, from day 0 to day 21 after inoculation. An increase on R_struct_biomass_ could already be observed at day 2 in all PCS, and in most cases reached a maximum at day 4. In PCS1 and PCS4 grown at 21°C the peak on R_struct_biomass_ is reached at day 7, since the speed (slope when a linear regression is fitted between two data points) of increase is slower.

### Expression of Carrot *AOX* Genes

#### PCS *de novo* Differentiation

The growth curve and aspect of callus along the 28 days of *in vitro* culture can be observed in **Supplementary Figure S1**. During the first 8 days in culture (lag-phase), growth of the explants was not visible. Then, exponential cell division started and callus proliferated until 28 days. During growth, callus lost the original orange color of the explants and progressively acquired a green color (**Supplementary Figure S1**).

**FIGURE 2:**
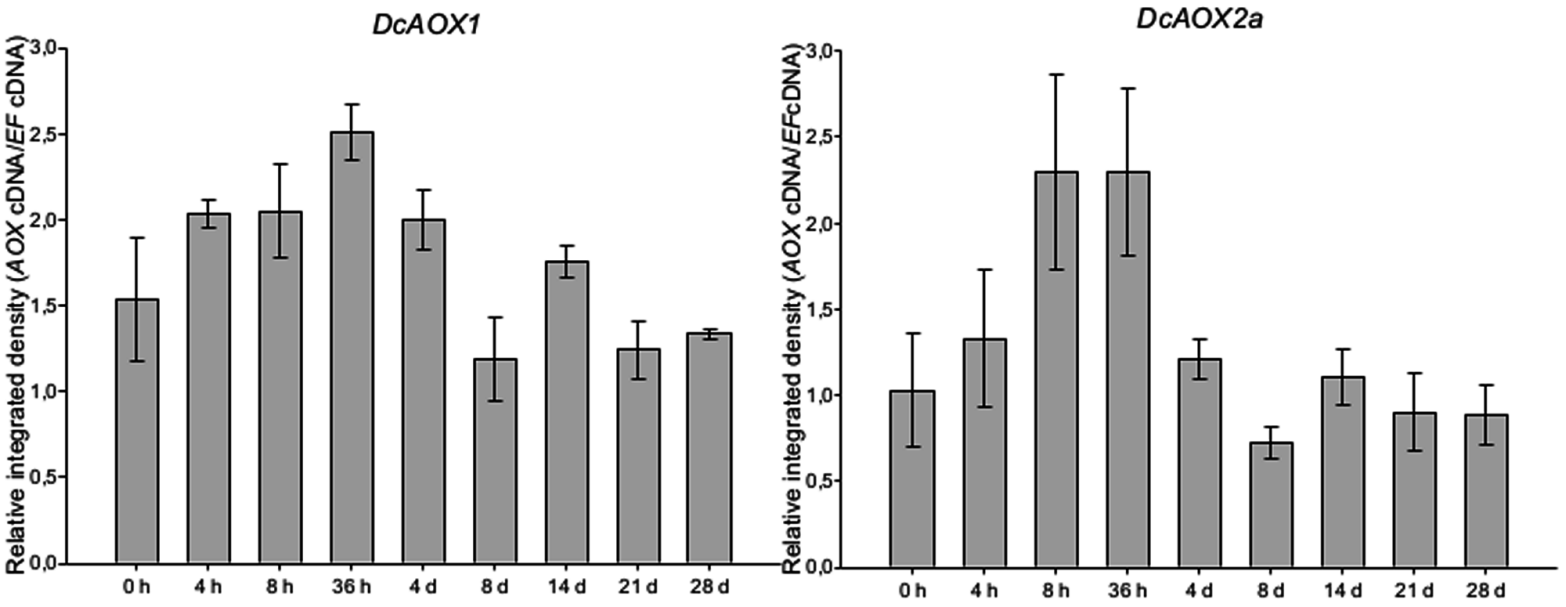
**Transcript levels of *DcAOX1* and *DcAOX2a* in primary cultures from secondary phloem of carrot roots.** Samples were collected at 0 h, 4 h, 8 h, 36 h, 4, 8, 14, 21, and 28 days post *in vitro* inoculation. Cultures were maintained at 21°C. Transcript levels were analyzed by RT-sqPCR using *DcEF1α* as reference gene. Normalization of the quantity of *DcAOX* transcripts was performed through the ratio of integrated densities *DcAOX* cDNA and *DcEF1α* cDNA bands. Data are the mean values ± SE of four individual plants.

Transcript levels for both *AOX* genes were found to slightly increase from the early beginning (4 hpi) of the lag-phase until 36 hpi (**Figure 2**). The time points showing highest level of transcripts (*P* > 0.05) were 4 hpi until 4 dpi for *DcAOX1*, and 8 hpi until 36 hpi for *DcAOX2a*. However, it was also observed that the timings of higher or lower expression were somewhat unphased between individual explants (not shown), thus reducing the possibility of observing significant differences between time points. At 4 dpi (lag phase), while the level of expression was still high for *DcAOX1, DcAOX2a* was already down regulated to values near the ones measured at 0 hpi. At the end of the lag phase and at initiation of exponential growth (8 dpi), expression of both genes achieved the lowest levels and remained relatively stable until 28 dpi, with values similar to the original, quiescent tissue (0 h). Expression patterns of *DcAOX1* and *DcAOX2a* significantly correlated (*P* = 0.01).

#### PCS Response to Temperature

**FIGURE 3:**
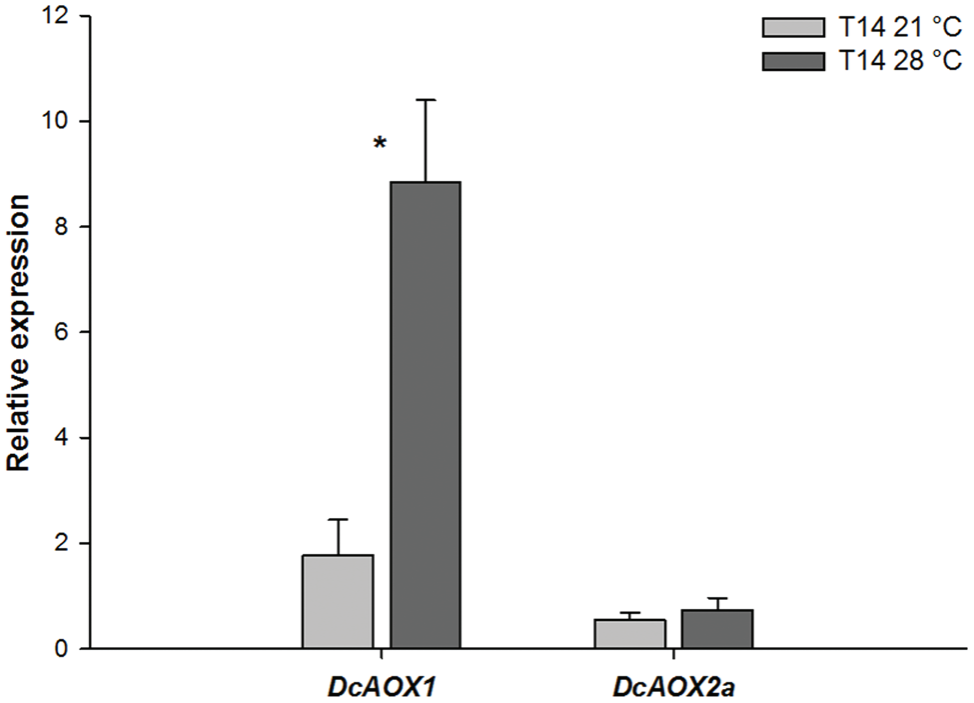
**Transcript levels of *DcAOX1* and *DcAOX2a* in primary cultures from secondary phloem of carrot roots grown at different temperatures.** The expression was normalized with *DcEF1α*. T14–21°C: explants after 14 days in culture, growing at 21°C; T14–28°C: explants after 14 days in culture, growing at 28°C. T0 (explants before inoculation) was used as calibrator. Data are the mean values ± SE of five individual plants. Student’s *t*-test was applied to test differences between temperatures for each gene. Significant differences are marked with ^∗^.

**Figure 3** shows expression levels for both *DcAOX* genes during exponential growth, at 14 dpi (T14), at two different growing temperatures (21°C and 28°C), in five explants with independent origins. *DcAOX1* was significantly higher expressed (fivefold) at 28°C than at 21°C (*P* < 0.05). No significant differences were observed for *DcAOX2a* between temperatures, and this gene showed low expression levels at both temperatures (**Figure 3**). These five plants had been selected for these studies on *AOX* gene expression variation from a larger group of 12 plants, because they showed variation in growth (not shown). Two showed significantly higher callus biomass at 28°C (R2 and R5), two had no significant differences between both temperatures (R1 and R3) and one showed a significantly higher callus FW at 21°C (R4). R3 was also characterized by low growth at both temperatures. However, concerning *AOX* transcript accumulation, at T14 during the exponential growth phase no direct link was detected between callus FW and individual *DcAOX1* or *DcAOX2a* transcript accumulation (not shown).

#### Plant Response to Chilling

**FIGURE 4:**
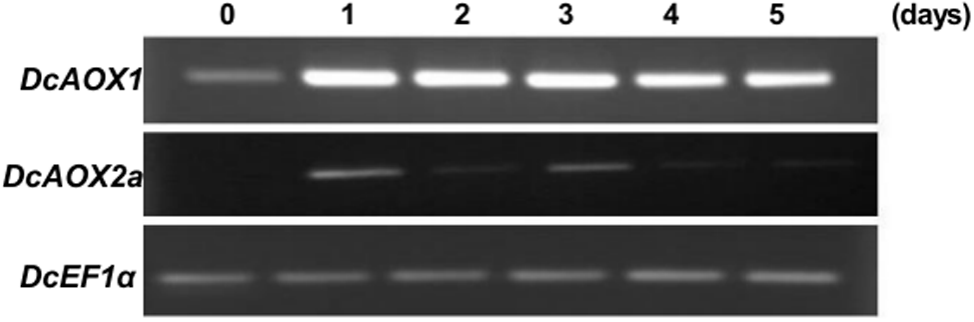
**Transcript level of *DcAOX1* and *DcAOX2a* genes in leaves of *Daucus carota* cv Rotin seedlings exposed to chilling (4°C) for 5 days.** *EF1α* cDNA was used as reference gene and the gel profiles are representative of three independent RT reactions from three biological replicates.

One month-old carrot seedlings exposed to 4°C for 5 days showed a similar induction pattern between both *DcAOX1* and *DcAOX2a* (**Figure 4**). The level of transcripts detected in *DcAOX1* was clearly higher than that of *DcAOX2a*. Expression levels of *DcAOX1* were high from day 1 to day 3 and decreased from day 4 to 5.

**FIGURE 5:**
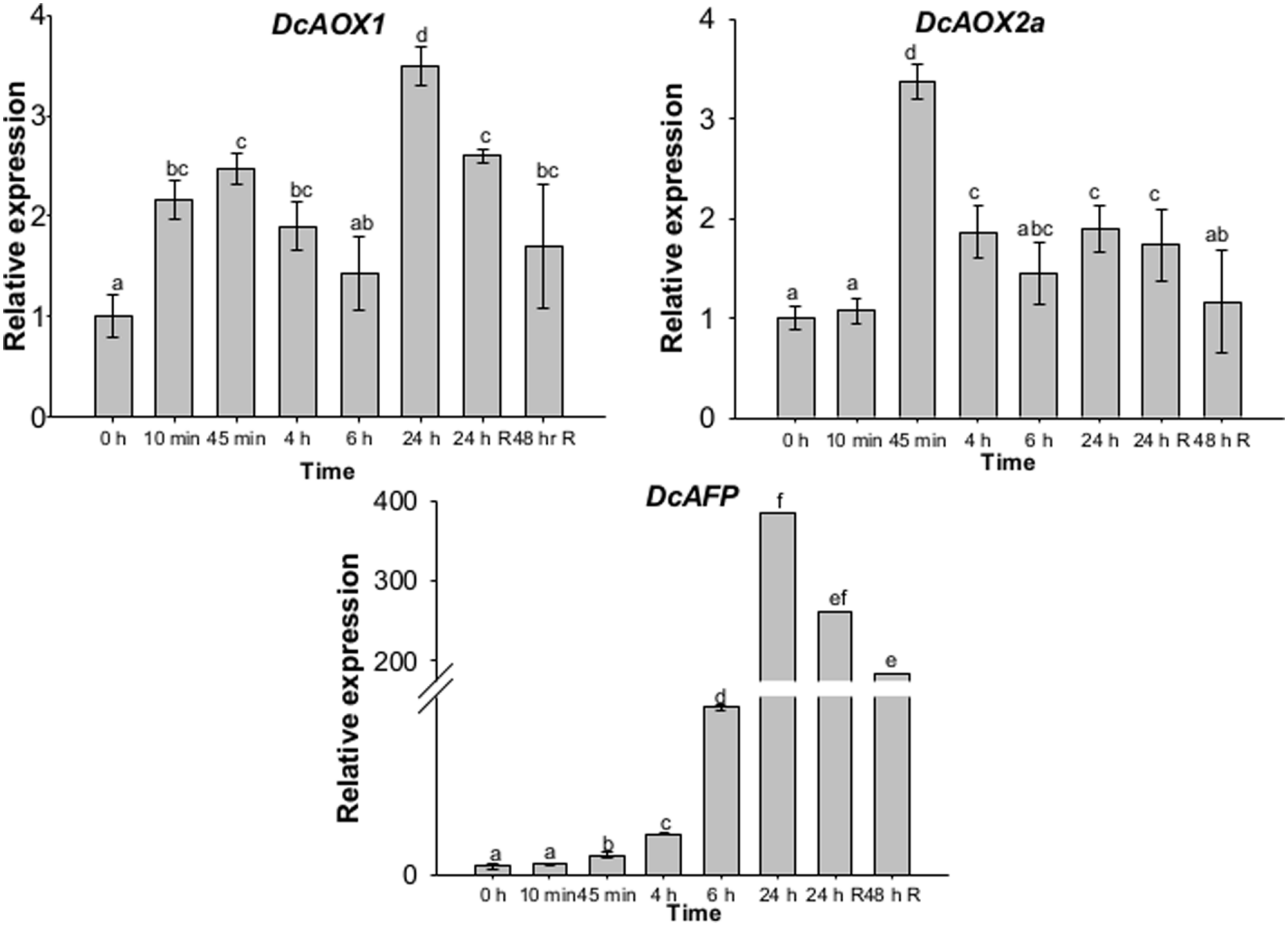
**Transcript levels of *DcAOX1, DcAOX2a*, and *DcAFP* during CE in 1-month-old carrot (*Daucus carota* L. cv. Rotin) seedlings.** Samples were harvested at 0 hpce (immediately before CE) and after 10 min, 45 min, 4, 6, and 24 h of exposure at 4°C and after 24 and 48 h after transferring plants back to the initial growth conditions (recovery period). Expression data was analyzed by RT-qPCR using *DcEF1α* as reference gene. Data are the mean values ± SE of four plants considered per time point. Statistical analysis (one-way ANOVA with Tukey’s *post hoc* test) was applied to each gene separately. Different superscript letters indicate significant differences between sampling points

When RT-qPCR analysis was performed with a higher time resolution until 24 hpce, both *AOX* genes showed very early responses to CE, since significantly higher mRNA levels were found at 45 min comparing to 0 hpce (*P* < 0.05) (**Figure 5**). In case of *DcAOX1*, an increase was observed immediately after 10 min of exposure with 2.2-fold difference in RE, followed by a 2.5-fold increment after 45 min. A slight transcript level reduction was observed, followed by a significant increase until 24 hpce (*P* < 0.05) (**Figure 5**). *DcAOX2a* increased 3.4-fold at 45 min of cold exposure relatively to 0 hpce (*P* < 0.05) (**Figure 5**). Transcript levels of *DcAOX2a* then decreased, showing constant levels until the 24 h of the recovery phase. By 48 h of recovery, a further reduction in mRNA levels was observed (**Figure 5**). Expression patterns of *DcAOX1* and *DcAOX2a* significantly correlated (*P* < 0.001).

Compared to both *AOX* genes, cold-responsive gene *AFP* in carrot showed a later but much higher level of transcripts in plants subjected to chilling stress. After 10 and 40 min of cold stress, the increase was only of 1.15 and 2.17-fold difference in RE from the 0 hpce, respectively, (**Figure 5**). However, *DcAFP* expression then highly increased, particularly at 24 hpce, showing an almost 400-fold difference comparing to 0 hpce (**Figure 5**).

### Analysis of Complete *DcAOX1* Sequences

A single band of expected size (ca. 450 bp) was obtained with primer combination P1/P2 and identified as *D. carota AOX1* based on high similarity with *AOX* gene sequences from other plant species available at NCBI database. Sequenced clones obtained were of 444 bp, and showed similarity between 75 and 99% with *AOX* from different plant species. For 5′-end isolation, reverse specific primer was used in combination with vector specific primer, which led to the amplification of a fragment near 1000 bp. For 3′-end isolation, the use of a forward specific primer in combination with the oligo d(T) primer, led to the amplification of fragments between 670 and 827 bp (described below). Based on match of 5′ and 3′-UTR sequences with initial partial exon 3 sequence, *in silico* full-length cDNA of *D. carota AOX1* (*DcAOX1*) was predicted.

**FIGURE 6:**
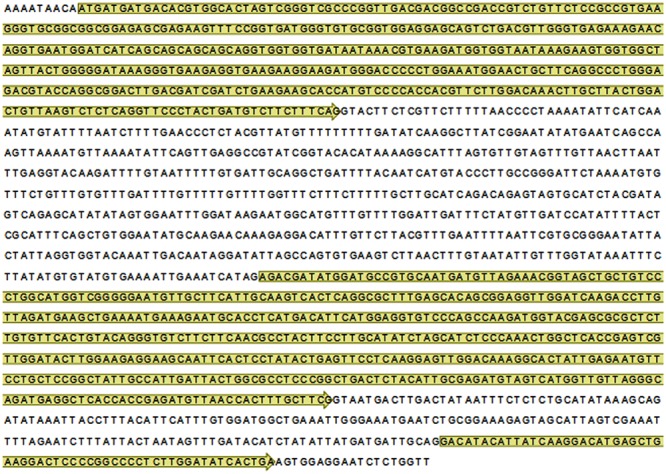
**Nucleotide sequence of gDNA encoding *Daucus carota* L. cv. Rotin *AOX1* (*DcAOX1)* (GenBank acc. n^o^. KJ669723).** Exons are indicated in yellow.

At genomic level, *DcAOX1* of *D. carota* L. cv. Rotin has 1812 bp, consisting of three exons (exon 1: 432 bp, exon 2: 489 bp, and exon 3: 57 bp) interrupted by two introns (intron 1: 630 bp and intron 2: 173 bp) (**Figure 6**). Gene structure of *DcAOX1* and structure of previously identified *AOX1* genes in several other plant species are shown in Supplementary Table S1. At transcript level, it has 1366 bp in length with a continuous open reading frame (ORF) of 981 bp, which encodes a putative polypeptide of 326 amino acid residues. The homologous identity score performed in NCBI with deduced amino acid residues showed that *DcAOX1* shares a high degree of similarity with AOX1 proteins from other plant species such as *Nicotiana benthamiana* (78%), *Brassica juncea* (73%), *Gossypium hirsutum* (72%), and *Arabidopsis lyrata* (70%). Different lengths of 3′-UTR were identified on *DcAOX1* cDNA sequences (3′ end isolation), varying between 185 and 344 bp (data not shown).

**FIGURE 7:**
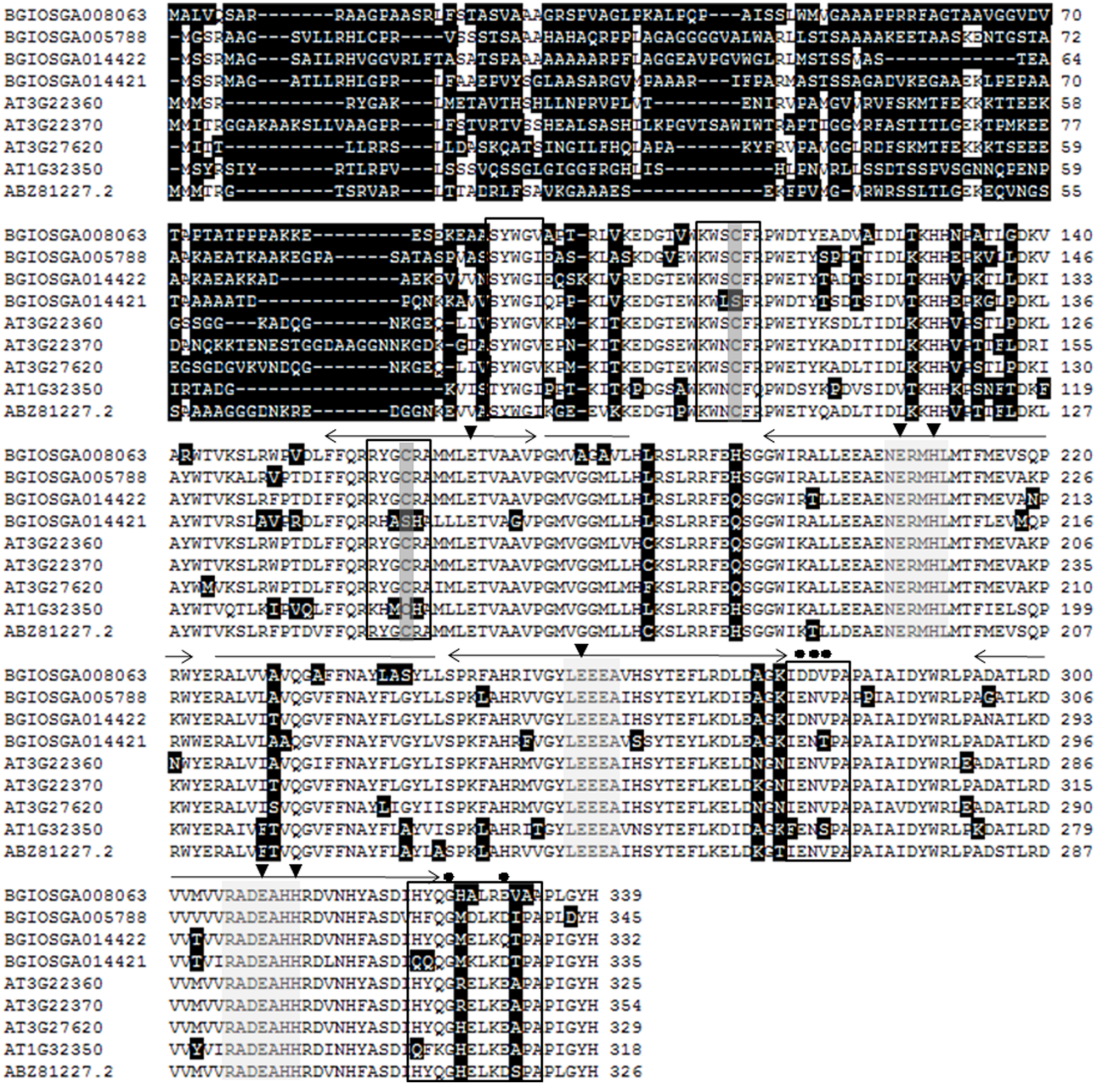
**Multiple alignment of translated amino acid sequences of previously reported AOX1 proteins from *Oryza sativa* (BGIOSGA008063, BGIOSGA005788, BGIOSGA14422, BGIOSGA14421), *A. thaliana* (AT3G22360, AT3G22370, AT3G27620, AT1G32350) (Cardoso et al., 2015) and DcAOX1 (ABZ81227.2).** Data retrieved from public web-based databases, freely available (Plaza: http://bioinformatics.psb.ugent.be/plaza/; e!EnsemblPlants: http://plants.ensembl.org/Multi/Search/New?db=core; and NCBI :http://www.ncbi.nlm.nih.gov/). Amino acid residues differing are shown on a black background, deletions are shown by minus signs. The sites of two conserved cysteins (CysI and CysII) involved in dimerization of the AOX protein by S–S bond formation (Umbach and Siedow, 1993) are indicated in dark gray boxes. Amino acid in light gray boxes are three regions defined by Berthold et al. (2000) as highly conserved in AOX. E (glutamate) and H (histidine) amino acid residues involved in iron-binding are indicated by filled triangles. Black boxes indicate the structural elements proposed to influence AOX regulatory behavior (Crichton et al., 2005), residues potentially involved in regulation of AOX activity are indicated by filled circles. Helical regions assumed to be involved in the formation of a hydroxo-bridged binuclear iron center (Andersson and Nordlund, 1999; Berthold et al., 2000) are shown by two-headed arrows above the amino acid sequences. Possible membrane-binding domains center (Andersson and Nordlund, 1999; Berthold et al., 2000) are shown with a line above amino acid sequences. The peptide sequences presented in this figure refer to the ORF translation of the sequences given in **Figure 6**.

DcAOX1 revealed structural features usually found in AOX1 sub-family members, with highly variable N-terminal region. DcAOX1 also showed two conserved cysteines (CystI and CystII) and di-iron-binding sites (**Figure 7**). DcAOX1 protein was predicted to be localized in mitochondria (mTP score of 0.868). The predicted length of the cleavage site of the mitochondrial targeting sequence is of 14 amino acids. Prediction of mitochondrial transit peptide for sequences used in the alignment of **Figure 7** shows no conservation across protein sequences and species, with DcAOX1 presenting the smallest predicted length. AOX1 sequences from *O. sativa* showed a predicted length of the mitochondrial targeting peptide of 67 (BGIOSGA008063), 58 (BGIOSGA005788), 54 (BGIOSGA014422), or 51 (BGIOSGA014421) amino acids. *A. thaliana* AOX1 predicted length of mitochondrial targeting peptide displayed 52 (AT3G22360), 63 (AT3G22370), 53 (AT3G27620), or 50 (AT1G32350) amino acids.

**FIGURE 8:**
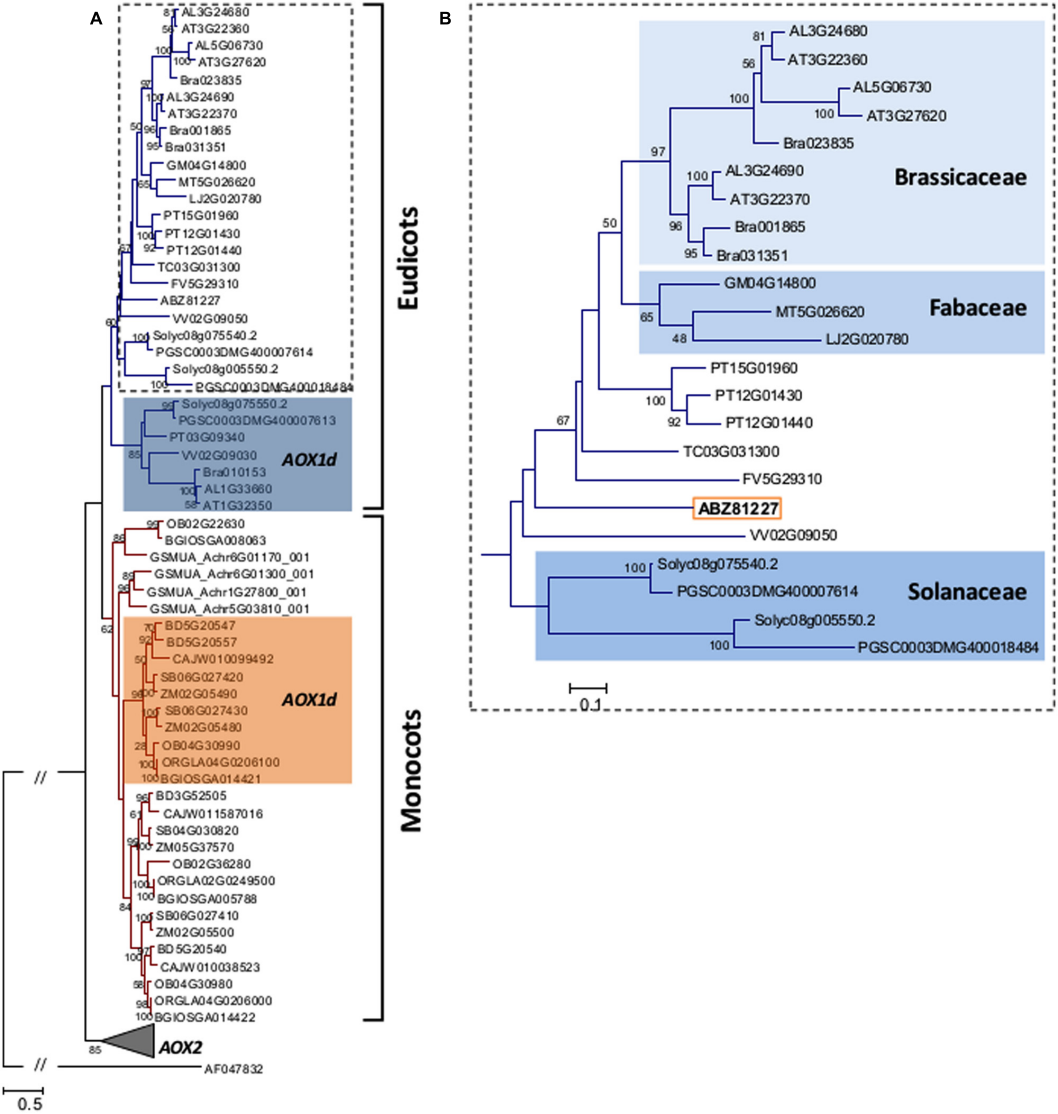
**(A)** Maximum likelihood (ML) tree showing the relationships among translated amino acid sequences of previously reported AOX1 proteins from plants, including the DcAOX1 sequence (ABZ81227.2) of *D. carota* L. cv. Rotin. Phylogeny reconstruction was done following the parameters described in the Section “Material and Methods”. Data retrieved from public web-based databases, freely available (Plaza: http://bioinformatics.psb.ugent.be/plaza/; e!EnsemblPlants: http://plants.ensembl.org/Multi/Search/New?db=core; and NCBI: http://www.ncbi.nlm.nih.gov/). *AOX* sequences were annotated by Cardoso et al. (2015) and *AOX1* sequences are identified in Supplemental Table S1. **(B)** AOX eudicot clade, without AOX1d representatives, showing the position of DcAOX1 sequence (ABZ81227.2).

The identified *D. carota* AOX1 sequence clearly nests within the AOX1 clade, and within the eudicots (**Figure 8**). Indeed, AOX1 sequences could be separated into two different groups: one including all eudicots sequences and other with the monocots (**Figure 8A**). Both within eudicots and monocots, the AOX1d clade was identified although not in an ancestral position (see Costa et al., 2014 for details). Within the eudicots, the Solanaceae, the Brassicaceae and the Fabaceae formed distinct monophyletic groups (**Figure 8B**).

## Discussion

### *AOX*, Cell Reprogramming, and Temperature-Dependent Growth

Cell reprogramming upon external stress initiates cascades of events including dedifferentiation and *de novo* differentiation (see Nagl, 1987, 1989, 1992; Arnholdt-Schmitt, 2004; Fehér, 2015 and references there in; Grafi and Barak, 2015). Dedifferentiation can be rapidly induced as shown by severe stress through protoplastation (within 24 h) (Fehér, 2015 and references therein). Since its beginning, plant tissue culture has substantially contributed to the current understanding of inducibility of differentiation events and the role of associated stress (e.g., Bassi, 1990; Arnholdt-Schmitt, 2001; Zavattieri et al., 2010; Grafi et al., 2011). The carrot PCS used for our experiments was first described by Steward et al. (1952) to study mechanisms of growth, and was later improved and maintained as an experimental system for studies on cell reprogramming (see review in Arnholdt-Schmitt, 1993b, 1999). In PCS, tissue dedifferentiation is induced in cells from the secondary phloem followed by callus growth initiation, mainly due to cell divisions (Arnholdt-Schmitt, 1993b). Cells from secondary phloem are quiescent adult cells that recently had developed by switching fate from meristem to phloem cells. The cambial root cells are considered target cells for both yield formation and environmental responses (Arnholdt-Schmitt, 1999). New meristem nests in the callus are unregularly distributed across the explant, beneath the periphery. Such cell fate switching can happen via stress-induced endogenous hormone regulation directly in perivascular stem cells, or indirectly via dedifferentiation in differentiated, competent cells, as it was shown for the well-studied process of somatic embryogenesis (e.g., Grieb et al., 1997).

The efficiency by which cell reprogramming can occur is of special interest, as this process is important for applied systems such as in breeding or commercial propagation. In a given system, efficiency might be limited during phase of induction or during initiation, or both. Fehér (2015) pointed that genetic differences for efficiency are more likely to be found during initiation. The usefulness of calorespirometry to study morphogenic responses (i.e., cell reprogramming) in *in vitro* cultures was first demonstrated by Kim et al. (2006) and later, this system was also used by Nogales et al. (2013) in *D. carota* cv. Rotin PCS to study temperature-dependent growth performance at 21°C and 28°C. In their study, an early peak around day 4 for R_struct_biomass_ was observed, which was coincident with the cell reprogramming process that happens in this system. In the present work, using this same system, we studied potential genotype differences on the early increase of R_struct_biomass_ associated with cell reprogramming process, and we found that the peak appeared homogeneously in all five tested PCS. However, the different slopes found in the curve of R_struct_biomass_ from day 0 to day 4 were indeed plant dependent. Genotype differences that can be measured by calorespirometry are supposed to differ in carbon use efficiencies, which in turn depend in plants essentially on the ratio of alternative and cytochrome oxidase respiration activity.

*AOX* genes seem to have a role during earliest events of cell reprogramming upon environmental changes (Arnholdt-Schmitt et al., 2006). For somatic embryogenesis, Frederico et al. (2009a) showed an early expression of *AOX* genes during initiation of the embryonic development (‘realization phase’ after depletion of auxin in the medium), being *DcAOX1* gene the one responding at higher level than *DcAOX2a*. In olive microshoots that were induced to rooting, callus growth that originated from cells close to the xylem and preceded rooting could not be inhibited by salicylhydroxamic acid (SHAM), a known inhibitor of AOX, while in the same system at the identical plants and morphologic region root induction was suppressed by SHAM (Macedo et al., 2012). This observation suggests that AOX is not required for callus growth *per se*. However, the induction of root organogenesis seemed to be linked to AOX activity. Therefore, a role for AOX may be seen in the early control of signaling and metabolic homeostasis for carbon and energy metabolism as a prerequisite for later balancing growth and development according to the available environmental conditions. This view confirms the proposal of Vanlerberghe (2013) and seems also to fit to the observations of Petrussa et al. (2009), who pointed the role of AOX as an anti-apoptotic factor in neighbor cells that have critical role for the reprogramming to somatic embryogenesis in *Abies alba* (see also Smertenko and Bozhkov, 2014; Arnholdt-Schmitt et al., 2015b). In carrot PCS, our results suggest a role of *DcAOX1* and *DcAOX2a* genes during the 1st hours of induced *de novo* differentiation of secondary phloem explants. These genes showed a modest though consistent increase in transcript levels until 36 h after inoculation and subsequent down-regulation before the beginning of the exponential growth. Due to the *a priori* unknown high intrinsic variability of the explants, future experiments must, however, include a higher number of samples.

AOX has been shown to be especially active in meristematic tissues (Hilal et al., 1997) and several studies have indicated links between AOX activity and plant growth (Arnholdt-Schmitt et al., 2006; Vanlerberghe, 2013 and references therein). Strong support for this view was found by experiments performed under various nutrient conditions in transgenic tobacco cells with silenced *AOX1a*. Sieger et al. (2005) demonstrated that *AOX1a*-knockdown led to the incapacity of the cells for down-regulating growth under P- and N-deficiency, and concluded that AOX activity provides a mechanism for adjusting growth and counteracting nutrient imbalance. When maintaining the knockdown of *AOX1a*, tobacco cell growth was connected to more stable carbon use efficiency. Arnholdt-Schmitt et al. (2006) also hypothesized on the importance of considering down-regulation of *AOX* as a potential tool for molecular breeding on higher nutrient efficiency. This led us to explore the hypothesis that differential *AOX* gene regulation relates to growth rates, with expected higher levels of *AOX* connected to suppressed growth or to lower growth rates, which indeed seems to fit to our observations during the lag phase of PCS growth, particularly until 8 days, where a dynamic behavior of both *AOX* genes but no increase in FW (although substantial amounts of nutrients were provided) were found. During lag phase, cells are thought to be prepared for the new fate and in PCS, first cells are reported to enter into the S-phase of cell cycling from 12 hpi to 24 hpi (Gartenbach-Scharrer et al., 1990). According to its known effect on cell homeostasis (Vanlerberghe et al., 2009; Vanlerberghe, 2013), AOX could have contributed to suppressing growth during lag phase.

Because temperature is a major environmental constraint and can influence the molecular mechanisms controlling growth, we have looked at the effect of temperature on *AOX* gene expression during exponential growth phase. Previous studies in PCS demonstrated that callus growth was significantly increased at 28°C compared to 21°C (Arnholdt-Schmitt, 1999), and our results showed that *DcAOX1* responded to a higher growing temperature in the exponential phase of the PCS. However no direct link was detected between callus FW and individual *AOX* genes expression (not shown).

In a first attempt to transpose these findings to plant level, we investigated both *AOX* genes in a chilling plant pot experiment, and compared it with the expression of the gene encoding the *AFP*. Interestingly, the two *AOX* genes were co-regulated in both PCS and pot experimental systems, which is in agreement with previous findings (Campos et al., 2009; Van Aken et al., 2009; Vanlerberghe, 2013). Clifton et al. (2005) analyzed the response of plant cells from *A. thaliana* at 3 h and up to 24 h post exposure upon various treatments designed to induce abiotic stress, and identified alternative respiration pathway components as the most important ones for early responses. The components of normal respiration were shown to be more stable during early times of acclimation without pronounced variations in transcript abundance. Low temperature stress – either chilling or freezing – is one of the most important abiotic stresses, with plants showing reduced yield (Beck et al., 2004). Our results indicated *DcAOX1* as the highest/rapidly responsive *AOX* gene during cold stress in carrot (**Figures 4** and **5**). Nevertheless, the patterns of transcript abundance over time course also revealed a further prevalent response of *DcAOX2a*, which was basal in control conditions (**Figure 4**). In general, *AOX1* is reported as a stress-responsive gene, whereas *AOX2* sub-family members were considered during long time as housekeeping genes or related to developmental events (Considine et al., 2002). Later on, *AOX2* members were found also to be involved in plastid-dependent signaling (Clifton et al., 2005) and in response upon several stress factors, including cold stress (Costa et al., 2010). *AOX2* stress response during chilling was also seen in the present study. In *A. thaliana*, Fiorani et al. (2005) reported a significantly lower leaf area in an *AOX1a* anti-sense line growing upon low temperature when comparing with the wild type. This phenotype was associated with reduced amount of *AOX* transcripts (almost entirely suppressed) and consequently low level of AOX protein. The authors suggested that the lower level of AOX1a protein could point to a reduced ability of the plant to cope with low temperature throughout the whole vegetative growth period. Taken these results together, it can be concluded that differential expression and co-regulation of diverse *AOX* family member genes might contribute to fine-tuning the metabolic-physiological cell responses upon stress toward deciding for growth and/or development.

### *DcAOX1* Sequence and Phylogenetic Analyses

Plant plasticity allows coping with stressful environmental conditions. Rapid acclimation and adaptation are desired plant characteristics, and target traits for which we aim to develop functional markers. It was thus our interest to look for genetic variability in a target gene, which could be linked with the desired trait. In this frame, the existence of polymorphisms in *AOX* gene sequences (alleles, haplotypes) is an essential basis for association studies to find links to achieve breeding goals (Arnholdt-Schmitt, 2015). Nevertheless, the measurement of the final effect of selected gene polymorphisms on complex traits such as abiotic stress tolerance must be performed following case-sensitive physiological approaches (Nogales et al., 2015). A system that involves the heterologous expression of AOX in the yeast *Schizasaccharomyces pombe*-that naturally does not have AOX genes (Albury et al., 1996) – is a potential candidate system for the study of the functionality of selected polymorphisms in *AOX* genes. The study of the effect of selected polymorphisms putatively linked to growth under different environmental conditions (e.g., high or low temperatures, salinity) can be aided by the use of specific technologies such as oxygraphy, stable oxygen isotope analysis and calorespirometry (Arnholdt-Schmitt et al., 2015a). With these technologies, AOX capacity, AOX *in vivo* activity and metabolic growth rates can be measured. This would enable finding the link between selected polymorphisms and the final effect on growth potential. These pre-screenings would help reducing significantly the number of plants to test in field trials to identify and validate candidate functional markers. Complete information about gene sequences is essential, since it is known that important/relevant differences between genotypes often occur not only in the coding region, but also in introns or UTRs. *DcAOX2a* gene isolation and variability in intronic regions among genotypes was already described (Campos et al., 2009; Cardoso et al., 2009). *DcAOX1* gene sequence and structural organization were still unknown and are here reported for the first time. However, although *AOX* genes could be general candidate markers related to diverse types of abiotic and biotic stress reactions, the role of AOX can differ between species and needs to be validated at species as well as at target tissue or cell level depending on the crop and breeding goals (Arnholdt-Schmitt, 2005, Arnholdt-Schmitt et al., 2006; Vanlerberghe, 2013). Future analyses on DcAOX protein content and activity will be essential for a better understanding of the involvement of DcAOX in response to stress conditions.

At transcript level, *DcAOX1* is 1366 bp in length, encoding a putative polypeptide of 326 amino acid residues. Variability on the *DcAOX1* 3′-end was observed, ranging from 185 to 344 bp, with 294 bp as the average size. The involvement of alternative polyadenylation (production of mature transcripts with 3′-ends of variable length) in oxidative stress response in plants has been pointed out (Xing and Li, 2011). *AOX* genes members present variability at the 3′-end, with a length ranging between 111 and 313 bp in maize (Polidoros et al., 2005) and between 76 and 301 bp in olive (Macedo et al., 2009). In *AOX1* genes, previous transcript analysis highlighted also the existence of length variation between *Arabidopsis* and *O. sativa* (Loke et al., 2005; Shen et al., 2008).

Analysis of the deduced amino acid sequence revealed structural features usually found in most of the higher plant AOXs (**Figure 6**). The role of some of these residues in AOX activity have been previously studied using site-directed mutagenesis in plants and other organisms such as protists, which revealed that many residues are critical for activity (Ajayi et al., 2002; Albury et al., 2002; Berthold et al., 2002; Crichton et al., 2005, 2010; Nakamura et al., 2005; Moore et al., 2013). Multiple sequence alignment showed a highly variable N-terminus of AOX1 sub-family as a result of low similarity amongst exon 1 sequences. Lack of homology in mitochondrial targeting signals is common and typical for proteins which require N-terminal signals for mitochondrial import (Finnegan et al., 1997). Despite the variability in length of the transit peptide, its presence is vital for targeting the peptide to mitochondria.

The conserved cysteine residues assumed to be involved in post-translational regulation of AOX activity (Umbach and Siedow, 1993; Rhoads et al., 1998; Grant et al., 2009) were also identified in DcAOX1. In some plant species, the conserved CysI in the N-terminal region of protein is replaced by SerI (Umbach and Siedow, 1993; Costa et al., 2009). This leads to regulation by succinate instead of pyruvate (Holtzapffel et al., 2003; Grant et al., 2009). Substitution of CysII by SerII can be observed in the alignment presented in the present work (**Figure 6**). However, physiological consequences of such changes are still unknown. Within AOX1 family monocots can be separated from eudicots and within the last ones, some groups form separated clades highlighting their common evolutionary history. Differential regulation on *AOX* gene subfamilies described by several authors may come from different positions in the plant genomes related to chromosomal territories (Arnholdt-Schmitt, 2004; Costa et al., 2009). The common distribution of *AOX* members is on at least two different chromosomes, at one gene per chromosome, occurring either in sense or antisense orientation (Cardoso et al., 2015). Furthermore, the presence of iron-binding motifs within four helical regions suggests that AOX might be a member of di-iron carboxylate protein family (Berthold et al., 2002; Berthold and Stenmark, 2003; Moore et al., 2008). Four conserved α-helical regions rich in histidine and glutamate were identified in *DcAOX1*, involved in the formation of hydroxyl bridged binuclear iron center (Siedow et al., 1995).

The predominant structure of genomic *AOX* sequences, including also both carrot *DcAOX2* genes (*DcAOX2a* and *DcAOX2b*), consists of four exons interrupted by three introns at well-conserved positions (Campos et al., 2009; Cardoso et al., 2015). Genes sharing this structure usually show exon size conservation for last three exons (Campos et al., 2009). Although not conserved, exon 1 presents a size around 300 bp (Campos et al., 2009; Cardoso et al., 2015). This feature is responsible for the formation of similar AOX proteins across different plant species. However, in *DcAOX1* loss of intron 1 was identified (Supplementary Table S1). Hence, a fusion of exon 1 and 2 could have consequently resulted in increase in exon size (432 bp) as compared to the common size of around 300 bp for exon 1 in genes showing the 4 exon structure. Nevertheless, it was observed that the last two exons showed a conserved size of 489 bp and 57 bp, respectively. Evolutionary intron loss and gain have resulted in the variation of intron numbers in some *AOX* members, with alterations in exons size (Considine et al., 2002; Polidoros et al., 2009; Cardoso et al., 2015; Supplementary Table S1). For instance, Cardoso et al. (2015) reported the existence of an *AOX* gene of *Oryza brachyantha* with six exons and five introns and showed the existence of an *Hordeum vulgare AOX* gene completely devoid of introns. Recent findings also showed the absence of introns in an *AOX* gene member of *Triticum urartu* (EnsemblPlants acc. n° TRIUR3_12374) (data not shown).

## Conclusion

With this study, calorespirometry arises as a suitable technology for the identification of cell reprogramming events related to metabolic and respiratory activity in carrot and shows a great potential to be used for phenotyping yield stability in *in vitro* systems. Our results are comparable with previous results showing an early peak in structural biomass formation during the lag phase of growth in the PCS, and show that *DcAOX1* and *DcAOX2a* were co-expressed in the earliest events in cell reprogramming upon environmental changes (inoculation or chilling). *DcAOX1* responded also to higher growing-temperature in the exponential phase of the PCS. For a better understanding of these results, the complete gene sequence of *DcAOX1* and its structural organization are analyzed. High-throughput genotype screening on complete *DcAOX1* and *DcAOX2a* genes could help on future identification of important within-gene motifs for co-regulation and differential transcript accumulation in view of novel resources for functional marker identification.

## Author Contributions

MC performed the PCS experiments and respective data processing and analysis, isolation of *DcAOX1* gene, and was driving writing of the manuscript. AN performed the calorespirometry measurements and respective data analysis. HC supervised RT-qPCR analysis, isolation of the *DcAOX1* gene and CE experiments. SRK performed the CE experiment and respective data processing. TN performed the phylogenetic analysis. RS was involved in CE experiment design. BA-S supervised responsibly AOX research, overall experiments design and writing of the manuscript. All authors read, critically revised and approved the manuscript.

## Conflict of Interest Statement

The authors declare that the research was conducted in the absence of any commercial or financial relationships that could be construed as a potential conflict of interest.

## Footnotes

1 http://imagej.nih.gov/ij/

2 www.macrogen.com

3 http://www.ncbi.nlm.nih.gov/

4 http://bioinformatics.psb.ugent.be/plaza/

5 http://plants.ensembl.org/Multi/Search/New?db=core

6 http://webblast.ipk-gatersleben.de/barley/

7 http://mafft.cbrc.jp/alignment/server/

8 http://www.ncbi.nlm.nih.gov/spidey/

## Supplementary Material

Figure S1 **(A)** Growth curve of primary cultures system of *D. carota* L. cv. Rotin during 28 days in culture at 21°C in four individual plants. Data are shown as callus FW values and represented as mean ± SD. **(B)** Explants from the secondary phloem of carrot tap roots PCS. Aspect of the explants (1) before (T0), (2) 14 days, and (3) 28 days after *in vitro* inoculation.

## Abbreviations

AFP: anti-freezing protein
AOX: alternative oxidase
cDNA: complementary DNA
CE: chilling exposure
*EF1α*: *Elongation factor 1 alpha*
FW: fresh weight
gDNA: genomic DNA
hpce/mpce/dpce: hours/minutes/days post chilling exposure
hpi/dpi: hours/days post inoculation
PCS: primary culture system
RE: relative expression
ROS: reactive oxygen species
RT-sqPCR: reverse transcription semi-quantitative real time polymerase chain reaction
RT-qPCR: reverse transcription quantitative real time polymerase chain reaction
