# Supplementary figures and images for: Stress-Induced Accumulation of DcAOX1 and DcAOX2a Transcripts Coincides with Critical Time Point for Structural Biomass Prediction in Carrot Primary Cultures (Daucus carota L.)
Source: Front Genet. 2016 Jan 29;7:1. doi: 10.3389/fgene.2016.00001 (PMC4731517; doi:10.3389/fgene.2016.00001)

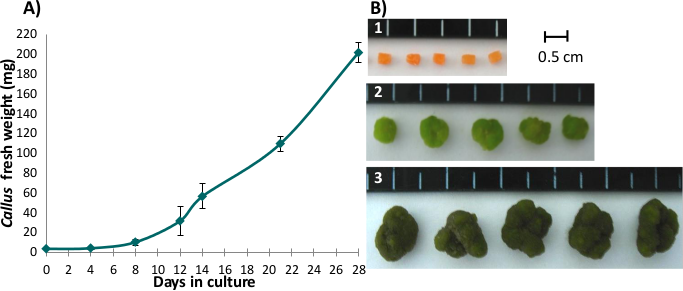

Supplement: Figure S1 — (A) Growth curve of primary cultures system of D. carota L. cv. Rotin during 28 days in culture at 21°C in four individual plants. Data are shown as callus FW values and represented as mean ± SD. (B) Explants from the secondary phloem of carrot tap roots PCS. Aspect of the explants (1) before (T0), (2) 14 days, and (3) 28 days after in vitro inoculation. [file Image_1.TIF]
