# Supplementary material for: Stress-Induced Accumulation of DcAOX1 and DcAOX2a Transcripts Coincides with Critical Time Point for Structural Biomass Prediction in Carrot Primary Cultures (Daucus carota L.)
Source: Front Genet. 2016 Jan 29;7:1. doi: 10.3389/fgene.2016.00001 (PMC4731517; doi:10.3389/fgene.2016.00001)
Supplement: Supplementary file 2 [file Table_1.DOC]

|  | ***Species*** | **Gene_id** | **Gene size** | **Protein length** | **Exon-intron (box-line) gene structure** 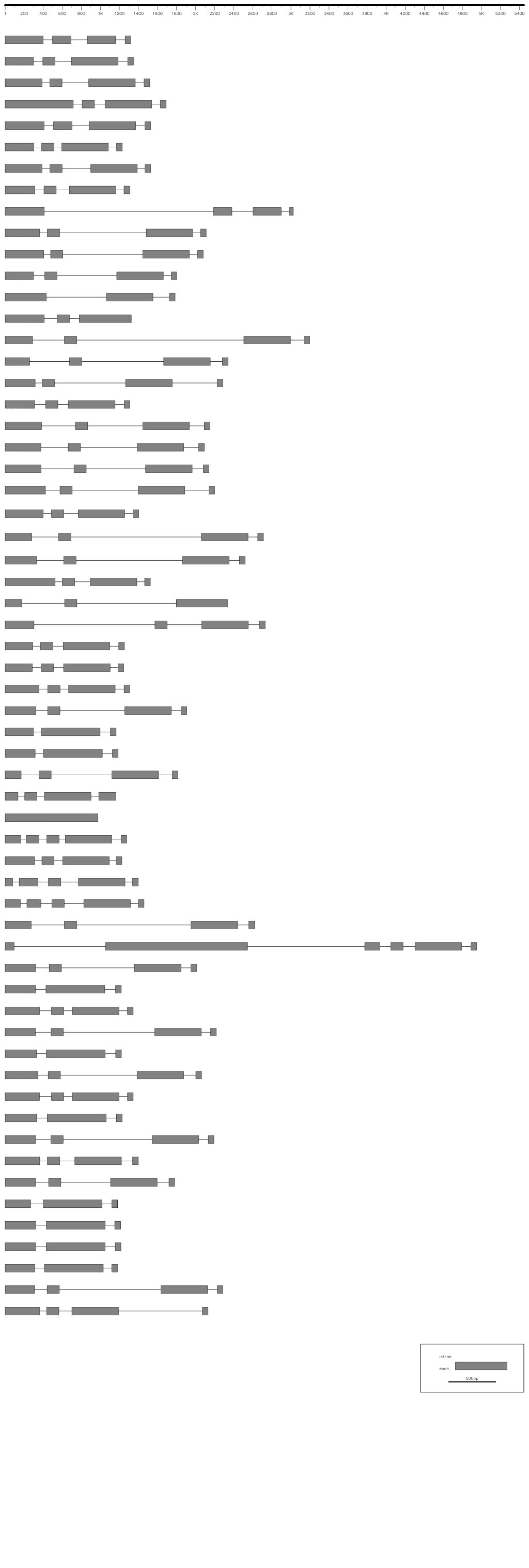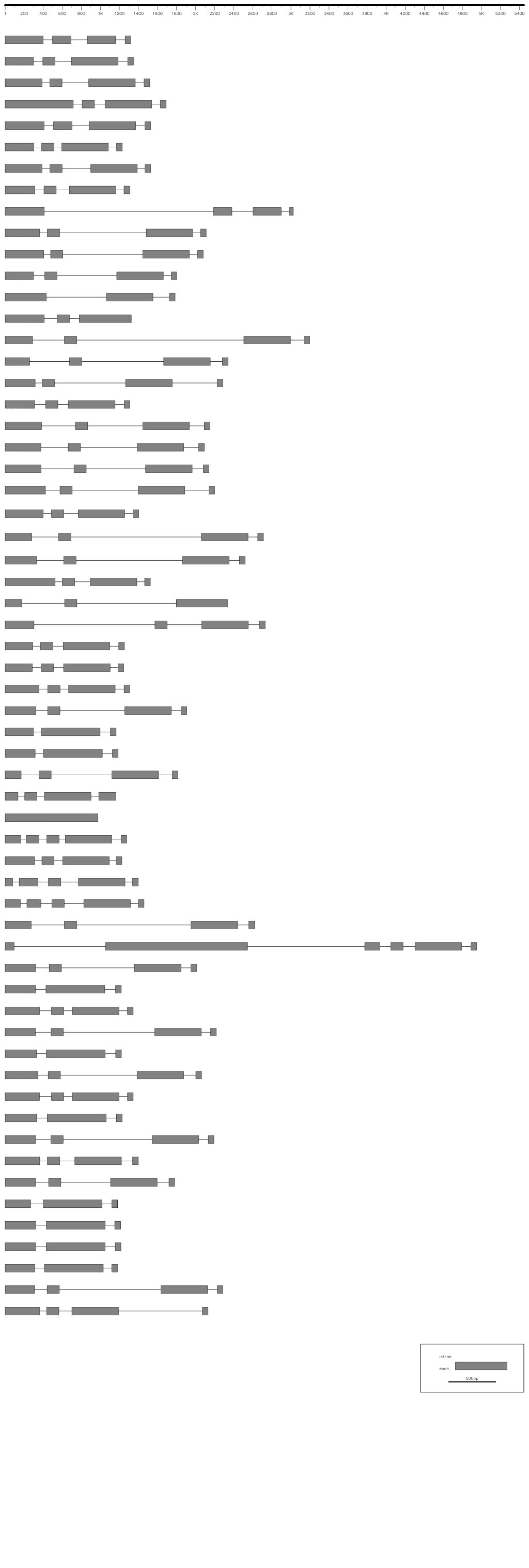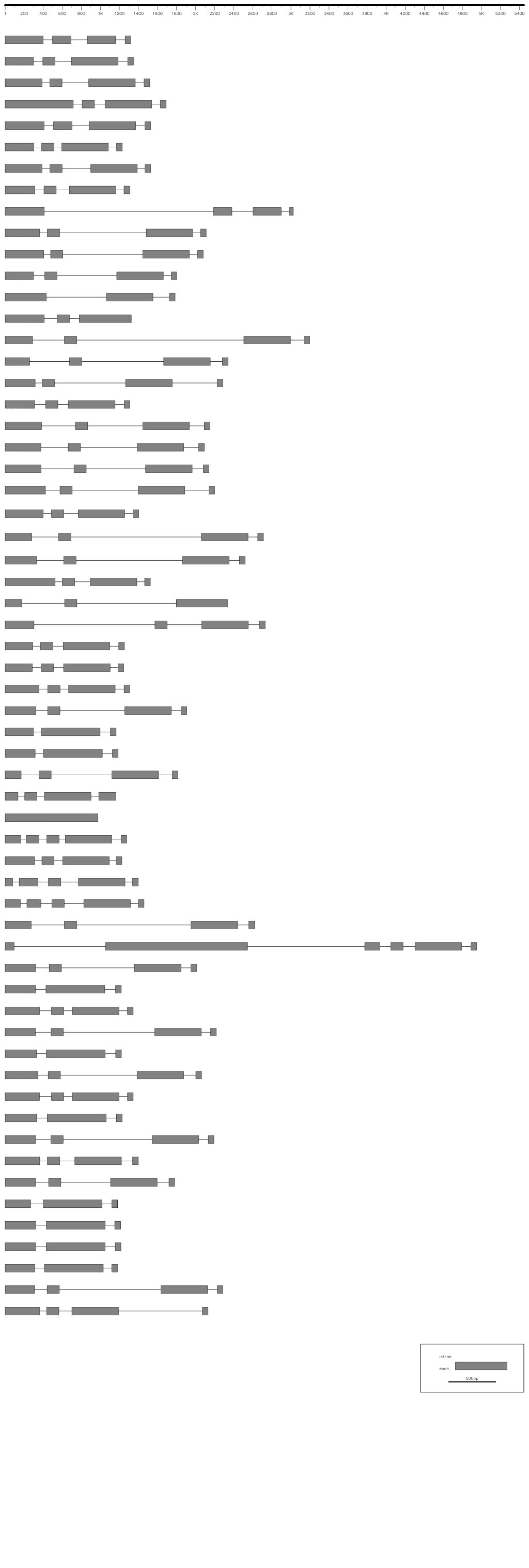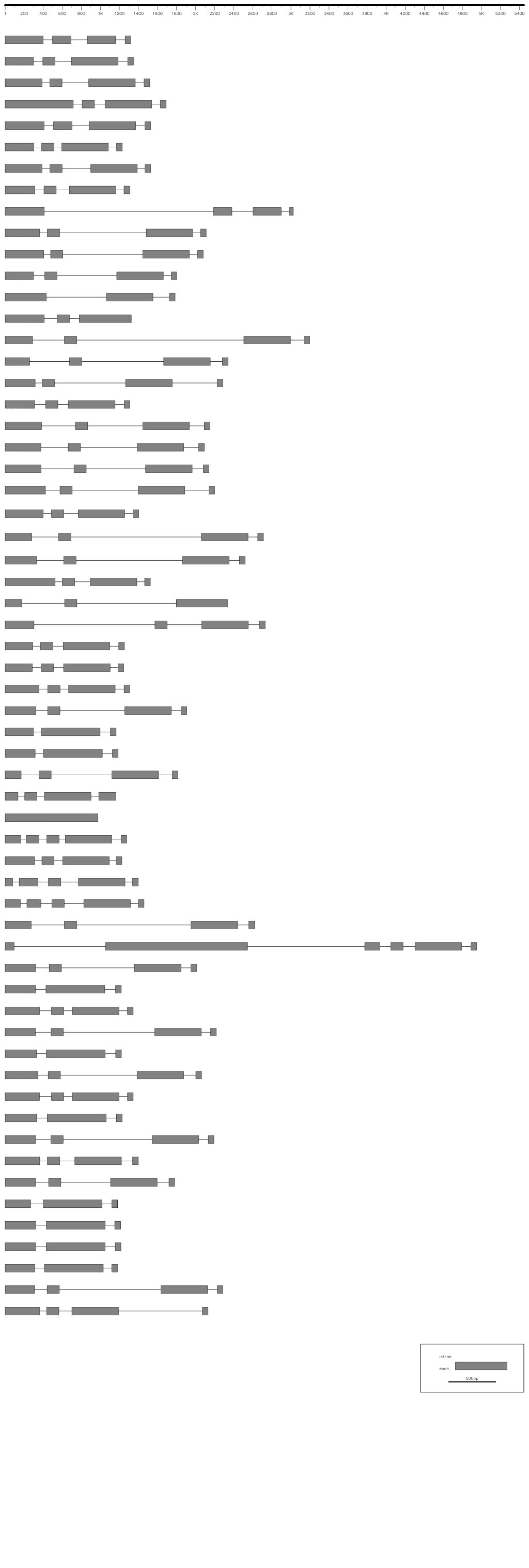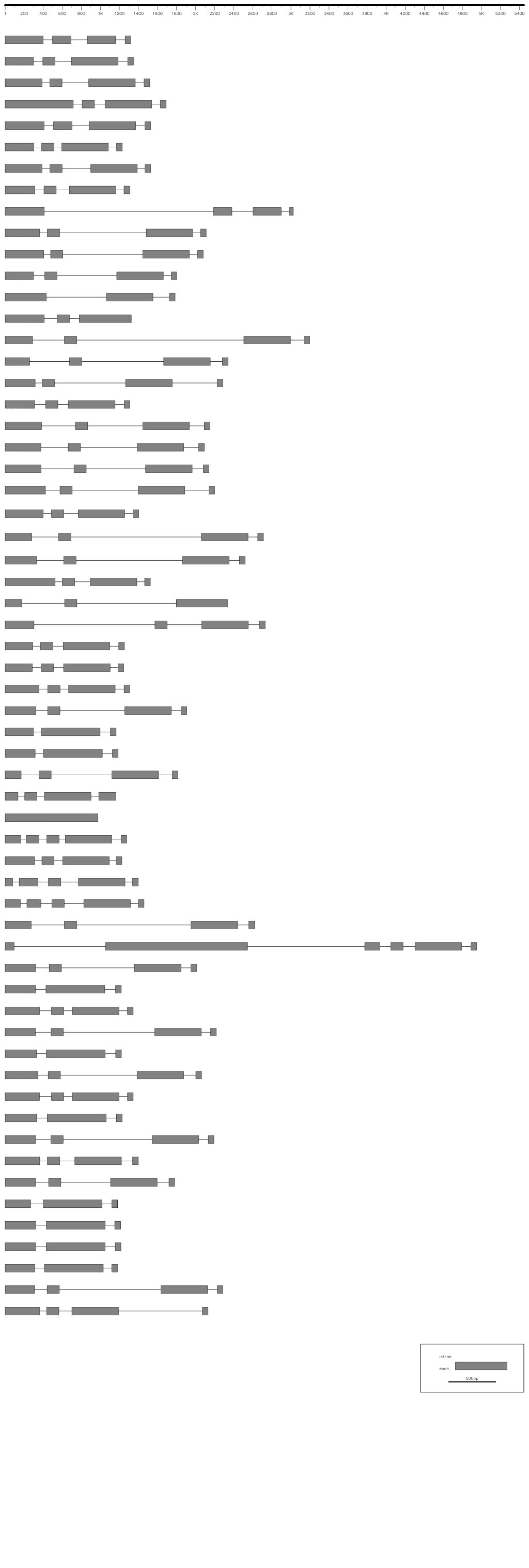 |
| --- | --- | --- | --- | --- | --- |
| ***Eudicots*** | *Arabidopsis lyrata* | AL1G33660 | 1319 | 315 |  |
|  |  | AL3G24680 | 1237 | 324 |  |
|  |  | AL3G24690 | 1518 | 354 |  |
|  |  | AL5G06730 | 1290 | 330 |  |
|  | *Arabidopsis thaliana* | AT1G32350 | 1333 | 318 |  |
|  |  | AT3G22360 | 1229 | 325 |  |
|  |  | AT3G22370 | 1527 | 354 |  |
|  |  | AT3G27620 | 1307 | 329 |  |
|  | *Brassica rapa* | Bra010153 | 3045 | 319 |  |
|  |  | Bra001865 | 2110 | 346 |  |
|  |  | Bra031351 | 2078 | 360 |  |
|  |  | Bra023835 | 1804 | 324 |  |
|  | *Daucus carota* | KJ669723 | 1366 | 326 |  |
|  | *Fragaria vesca* | FV5G29310 | 1323 | 361 | **Δ** |
|  | *Glycine max* | GM04G14800 | 3196 | 321 |  |
|  | *Lotus japonicus* | LJ2G020780 | 2471 | 314 |  |
|  | *Medicago truncatula* | MT5G026620 | 2287 | 330 |  |
|  | *Populus trichocarpa* | PT03G09340 | 1310 | 329 |  |
|  |  | PT12G01430 | 2149 | 352 |  |
|  |  | PT12G01440 | 2091 | 350 |  |
|  |  | PT15G01960 | 2140 | 351 |  |
|  | *Solanum lycopersicum* | Solyc08g005550.2 | 2198 | 366 |  |
|  |  | Solyc08g075540.2 | 1402 | 358 |  |
|  |  | Solyc08g075550.2 | 2711 | 318 |  |
|  | *Solanum tuberosum* | PGSC0003DMG400007613 | 2518 | 321 |  |
|  |  | PGSC0003DMG400007614 | 1524 | 356 |  |
|  |  | PGSC0003DMG400018484 | 2333 | 279 |  |
|  | *Theobroma cacao* | TC03G031300 | 2729 | 326 |  |
|  | *Vitis vinifera* | VV02G09030 | 1252 | 322 |  |
|  |  | VV02G09050 | 1245 | 320 |  |
| ***Monocots*** | *Brachypodium distachyon* | BD3G52505 | 1308 | 343 |  |
|  |  | BD5G20540 | 1907 | 333 |  |
|  |  | BD5G20547 | 1165 | 324 | ⚫ |
|  |  | BD5G20557 | 1186 | 330 | ⚫ |
|  | *Hordeum vulgare* | CAJW010038523 | 1815 | 281 |  |
|  |  | CAJW011587016 | 1163 | 270 |  |
|  |  | CAJW010099492 | 975 | 324 | ▲ |
|  | *Musa acuminata* | GSMUA_Achr5G03810_001 | 1277 | 324 | ♦ |
|  |  | GSMUA_Achr6G01170_001 | 1225 | 328 |  |
|  |  | GSMUA_Achr6G01300_001 | 1397 | 317 | ♦ |
|  |  | GSMUA_Achr1G27800_001 | 1458 | 327 | ♦ |
|  | *Oryza brachyantha* | OB02G22630 | 2617 | 316 |  |
|  |  | OB02G36280 | 4950 | 806 |  |
|  |  | OB04G30980 | 2009 | 331 |  |
|  |  | OB04G30990 | 1219 | 331 | ⚫ |
|  | *Oryza glaberrima* | ORGLA02G0249500 | 1344 | 345 |  |
|  |  | ORGLA04G0206000 | 2216 | 331 |  |
|  |  | ORGLA04G0206100 | 1220 | 335 | ⚫ |
|  | *Oryza sativa* | BGIOSGA008063 | 2061 | 339 |  |
|  |  | BGIOSGA005788 | 1344 | 345 |  |
|  |  | BGIOSGA014421 | 1227 | 335 | ⚫ |
|  |  | BGIOSGA014422 | 2191 | 332 |  |
|  | *Sorghum bicolor* | SB04G030820 | 1398 | 346 |  |
|  |  | SB06G027410 | 1779 | 331 |  |
|  |  | SB06G027420 | 1179 | 314 | ⚫ |
|  |  | SB06G027430 | 1210 | 332 | ⚫ |
|  | *Zea mays* | ZM02G05480 | 1215 | 332 | ⚫ |
|  |  | ZM02G05490 | 1178 | 329 | ⚫ |
|  |  | ZM02G05500 | 2287 | 329 |  |
|  |  | ZM05G37570 | 2136 | 347 |  |

**Supplemental Table 1** Diversity of *AOX1* in exon-intron pattern across higher plants. Data retrieved from public web-based databases, freely available (Plaza: <http://bioinformatics.psb.ugent.be/plaza/>; e!EnsemblPlants: <http://plants.ensembl.org/Multi/Search/New?db=core>; IPK Barley Blast Server: <http://webblast.ipk-gatersleben.de/barley/>; NCBI: http://www.ncbi.nlm.nih.gov/). Gene draw was performed in FancyGene 1.4 (Rambaldi and Ciccarelli, 2009). (adapted from Cardoso et al., 2015).

- loss of intron 1, **Δ** loss of intron 3, ⚫ loss of intron 2, ▲ loss of all introns, ♦ gain of intron in exon 1
